# A novel phage vB_VhaM_HVS-1 inhibits host cell associated *Vibrio harveyi*

**DOI:** 10.3389/fvets.2026.1908714

**Published:** 2026-08-31

**Authors:** Qun Chen, Peipei Ye, Jieting Pan, Lin Chen, Xibang Liu, Lijian Ding, Liming Jiang

**Affiliations:** 1 Department of Respiratory and Critical Care Medicine, The Affiliated Yangming Hospital of Ningbo University (Yuyao People’s Hospital), Ningbo University, Ningbo, Zhejiang, China; 2 Ningbo Key Laboratory of Malignant Tumor Immunodiagnosis and Immunotherapy, Health Science Center, Ningbo University, Ningbo, Zhejiang, China; 3 School of Basic Medical Sciences, Health Science Center, Ningbo University, Ningbo, Zhejiang, China; 4 Department of Hematology, The Affiliated People’s Hospital of Ningbo University, Ningbo, Zhejiang, China; 5 Institute of Hematology, Ningbo University, Ningbo, Zhejiang, China; 6 Ningbo No. 2 Hospital, Wenzhou Medical University, Ningbo, Zhejiang, China; 7 School of Pharmacy, Health Science Center, Ningbo University, Ningbo, Zhejiang, China; 8 School of Pharmacy, Changsha Medical University, Changsha, China

**Keywords:** aquaculture disease, bacteriophage, coelomocyte protection, lytic virus, sea cucumber vibriosis

## Abstract

**Introduction:**

*Vibrio harveyi* is a major pathogen causing skin ulceration syndrome and mass mortality in sea cucumber *Apostichopus japonicus* aquaculture.

**Methods:**

In this study, a pathogenic strain NB-3 was isolated from diseased *A. japonicus* and identified as *V. harveyi* based on morphological, physiological, and genomic analyses. A novel lytic phage vB_VhaM_HVS-1 belonging to the family *Straboviridae* (class *Caudoviricetes*) was isolated from *A. japonicus* breeding pond silt.

**Results:**

The phage exhibited a latent period of 50 min and a burst size of approximately 168 PFU/cell, and its genome lacked lysogeny-related genes, suggesting a lytic nature. Phage treatment significantly reduced the number of cell-associated bacteria, decreasing the association rate from 2.3% to nearly 0. Flow cytometry revealed that phage infection protected coelomocytes from *V. harveyi*-induced damage, increasing the proportion of viable cells from 78.17% to 88.54%.

**Conclusion:**

These findings demonstrate that phage vB_VhaM_HVS-1 effectively reduces *V. harveyi* association with coelomocytes and alleviates host cell apoptosis. The phage shows strong potential as an alternative biocontrol agent against *V. harveyi* infection in sea cucumber aquaculture.

## 1 Introduction

The genus *Vibrio* is one of the most common bacterial groups in brackish water and marine environments. They are widely distributed in the coastal waters, estuaries, colonise marine organisms and aquatic ecosystems, which are Gram-negative bacterium with a short body and a curved tail with a flagellum (1–3). *Vibrio spp*. have been implicated as aetiological agents of disease in sea cucumber, shrimp, fish and shellfish (4). Among them, the Harveyi clade, which includes *Vibrio campbellii* and *Vibrio harveyi*, contains closely related species that cause disease in both cultured and wild aquatic organisms (5, 6). *V. harveyi* is a facultatively anaerobic, motile, rod-shaped, halophilic Gram-negative bacterium that exhibits characteristic bioluminescence regulated by quorum sensing. It grows optimally at 25–30 °C and salinity of 15–40‰, thriving in diverse marine environments (7), and often carries resistance genes to ampicillin, streptomycin, and tetracycline, complicating antibiotic therapy (8). In aquaculture, it is the primary agent of luminescent vibriosis, causing mass mortality in shrimp, fish, and bivalves.

Bacteriophages (phages) are viruses that specifically infect bacteria, spirochetes, and actinomycetes (9). They are highly host-specific and do not infect humans or animals, making them promising antibacterial agents that can serve as alternatives to antibiotics (10). With the increasing problem of antibiotic resistance, there is an urgent need for phage-derived products or phages as alternatives (11, 12). Several phages targeting Harveyi clade bacteria have been isolated and characterized. Srisangthong et al. isolated and characterized a *Siphoviridae* family lytic *V. campbellii* phage OPA17 from bloody clams, and found that the addition of phage OPA17 significantly increased the survival of *Artemia franciscana* nauplii infected with *V. campbellii* (13). Li et al. reported a novel *Caudovirales* phage vB_VcaS_HC containing a lysogeny-related gene, which nevertheless exhibited strong lytic activity against pathogenic *V. campbellii* in aquaculture seawater, highlighting the importance of genomic safety screening (14). More recently, Moon et al. characterized three novel phages from Korean shrimp hatchery wastewater, among which vB_VhaS-MS03 maintained effective bacterial control even at an extremely low MOI of 0.01 (15). Despite these promising developments, the emergence of phage-resistant bacteria remains a critical challenge for phage therapy. Resistance can arise through mutations in bacterial surface receptors that phages recognize for adsorption, as well as through CRISPR-Cas adaptive immunity systems (16). To mitigate resistance, phage cocktails targeting multiple receptors or rotation strategies have been proposed as effective approaches (17). From a translational perspective, several phage-based products have already entered the aquaculture market. For example, commercial phage products targeting *Vibrio* species in shrimp hatcheries have demonstrated significant reductions in mortality (18), and phage-based biocontrol agents for finfish and shellfish are under active development (19). These advances underscore the growing practical potential of phage therapy in aquaculture settings.

Apoptosis is an essential biological process that plays crucial roles in cellular and tissue homeostasis, embryonic development, and immunity (20, 21). In multicellular organisms, apoptosis mediates the immune defense against the invading parasites, bacteria and viruses by eliminating the infected, damaged, unstable and needless cells (22, 23). The sea cucumber *A. japonicus* is a kind of representative economic echinoderm species in the Bohai Sea and Yellow Sea of China. *V. harveyi* has a wide range of hosts, caused serious economic losses, and widely threatens the sustainable development of this *A. japonicus* industry (24). Various pathogens, including *Vibrio, Pseudomonas,* and even viruses leading the skin ulceration syndrome of *A. japonicus*, results in 90–100% mortality. As a marine invertebrate, *A. japonicus* lacks the acquired immune system and its innate immunity mainly depends on immune factors and coelomocytes to respond to pathogen invasion (25). Modulation of apoptosis is considered an active innate immune mechanism during pathogen infection, and components of Gram-negative bacteria, such as lipopolysaccharide (LPS), have been shown to influence this process (26–30).

In this study, we isolated and characterized a novel lytic phage, vB_VhaM_HVS-1, evaluated its ability to inhibit *V. harveyi* adhesion to coelomocytes and protect against *V. harveyi* induced coelomocyte apoptosis in *A. japonicus*. These findings suggest that phage has potential as an alternative biocontrol agent for *V. harveyi* infection in sea cucumber aquaculture.

## 2 Materials and methods

### 2.1 Bacteria isolation and growth conditions

*V. harveyi* was isolated from *A. japonicus* with skin ulceration syndrome. The diseased sea cucumbers were kept in oxygenated bags at 0 °C and transported to the Ningbo university laboratory for pathogen isolation and diagnosis. Prior to bacterial isolation, the *A. japonicus* surface was disinfected with 75% ethanol and aseptically dissected. Samples of body wall, muscle, tentacle, respiratory tree, and intestine were collected and streaked directly onto 2216E agar plates. The plates were incubated at 28 °C for 24 h for the bacterial colonies to grow. A single dominant bacterial colony was selected and streaked on the 2216E media. Representatives of one selected bacterial colonies, designated NB-3, was purely sub-cultured, suspended in 2216E containing 15% (v/v) glycerol at −80 °C. The GFP-tagged *V. harveyi* NB-3 strain was constructed by electroporation with a GFP-expressing plasmid (pGFPuv, Clontech) and selected on 2216E agar plates containing ampicillin (100 μg/mL). GFP expression was confirmed by fluorescence microscopy.

### 2.2 Genome sequencing and analysis

Genomic DNA of the *V. harveyi* NB-3 was extracted using the magnetic beads method, lysozyme and protease are combined to decompose and digest, and purified DNA was adsorbed by binding solution and magnetic beads. Fragmented gDNA was used for library construction with a Hieff NGS® MaxUp II DNA Library Prep Kit for Illumina® (Shanghai Yisheng Biotechnology Co., Ltd., 12200ES08) and sequenced on the Illumina HiSeq platform (Sangon Biotech, China). Raw reads were quality-filtered and assembled using SPAdes assembler. Gene prediction and functional annotation were performed using the NCBI Prokaryotic Genome Annotation Pipeline (PGAP), and the genome sequence was deposited in GenBank under the accession number PZ791701.

### 2.3 Phylogenetic analysis

The 16S rRNA sequence predicted from the genome was compared against the NCBI 16S rRNA database using BLAST with an identity threshold of >95%. The top 30 sequences with the highest identity were selected, and a phylogenetic tree was constructed using multiple sequence alignment followed by FastTree software.

### 2.4 Phage isolation and purification

The phage was isolated from silt samples collected from *A. japonicus* breeding pond in Dalian, China. The propagation method was modified from Park (31). Briefly, 3 g of a *A. japonicus* breeding pond silt sample was mixed with 3 mL phosphate buffer solution in a 10 mL centrifuge tube and incubated with shaking at 180 rpm for 1 h at room temperature. Then, the sample was centrifuged at 6,000 × g for 6 min, and the supernatants were filtered with a 0.22 μm filter membrane. After filtration, 3 mL of each filtrate was inoculated onto log phase-grown *V. harveyi* in 20 mL of 2216E culture broth and incubated for 48 h. Then, the culture was centrifuged at 6000 × g for 6 min, and the supernatant was filtered with a 0.22 μm filter membrane. The filtrate was serially diluted 10 times, mixed with 5 mL molten 0.45% 2216E soft agar containing *V. harveyi* (2 × 10^8^ CFU/mL), and immediately added to a 2216E plate. The growth of overnight cultures and plaque formation were observed after 12 h. A single phage plaque was selected for phage purification, and the process was repeated three times.

### 2.5 Growth curve and adsorption rate of the isolated phage

To measure the adsorption rate, the co-culture of phage vB_VhaM_HVS-1 (1 × 10^8^ PFU/mL) and logarithmic phase *V. harveyi* were mixed at an MOI of 0.1 and cultured at 28 °C; the phage titer was measured after 0, 5, 10, 15, 20, 25 and 30 min. Free phages were measured by titrating the supernatant after centrifugation at 6,000 × g for 5 min at 4 °C. The one-step growth curve of phage vB_VhaM_HVS-1 was carried out as follows. Briefly, 20 mL of exponential phase *V. harveyi* culture was harvested by centrifugation (6,000 g, 5 min, 4 °C), and the pellet was resuspended in 20 mL of fresh 2216E to obtain an OD_600_ of 1.0. Next, 20 mL of phage vB_VhaM_HVS-1 was added to reach an MOI of 1 and allowed to adsorb for 15 min at 28 °C. The mixture was centrifuged at 6,000 × g for 5 min at 4 °C, and the pellet was resuspended in 50 mL of fresh 2216E. At each time point, samples were taken and immediately plated to quantify released phages. Samples were taken every 10 min for 120 min, after which the supernatants were plated on 2216E agar to determine the phage titer. The number of infected cells was estimated from the initial bacterial concentration and phage input at MOI = 1. Burst size was calculated as the ratio of the final phage titer at plateau to the initial titer after adsorption. For the adsorption assay, free phages were measured by titrating the supernatant. All experiments were performed in triplicate (*n* = 3). *V. harveyi* revived in 2216E medium broth with 4‰ (V/V), cultured at 28 °C for 12 h, then the same vaccination rate 4‰ (V/V) inoculated in fresh 2216E, cultured at 28 °C, at the same time, added phage for culture according to the multiple of infection MOI = 0.1 and 1. The optical density (OD_600_) was measured every 1.5 h over a 9 h period using a spectrophotometer.

### 2.6 TEM analyses of the isolated phage

The morphology of the phage vB_VhaM_HVS-1 particles was analyzed using transmission electron microscopy (TEM) mainly with the steps described below. Dilutions of the phage vB_VhaM_HVS-1 stock (approximately 5 × 10^9^ PFU/mL) were deposited on carbon film and stained with 2% uranyl acetate. Phage vB_VhaM_HVS-1 samples were observed using a Philips EM 300 electron microscope operated at an acceleration voltage of 120 kV at Ningbo University (Ningbo, China). Phage vB_VhaM_HVS-1 was identified and classified according to the International Committee on Taxonomy of Viruses.

### 2.7 Sequencing and analysis

Purified phage vB_VhaM_HVS-1 was concentrated through a 10 kDa filter and treated with DNase and RNase at 37 °C for 1 h. A Takara Minibest Viral RNA/DNA Extraction Kit (Cat#9766) was used to extract phage vB_VhaM_HVS-1 genomic DNA. Sequencing of the phage vB_VhaM_HVS-1 genomic DNA was carried out on the Illumina HiSeq platform (Sangon Biotech, China). The resulting reads were assembled using SPAdes assembler (v3.15.5) with default parameters, and quality control was performed using FastQC software. Open reading frames (ORFs) were predicted using Prokka (v1.14.6) and annotated using NCBI BLAST+ (v2.13.0) searches against multiple databases, including TrEMBL, KOG, COG, CDD, NT, PFAM, SwissProt and NR, to obtain functional annotation information for the phage vB_VhaM_HVS-1 gene protein sequences. Additionally, targeted screening for virulence genes, toxin genes, and antimicrobial resistance genes was performed using VFDB (Virulence Factors Database) and CARD (Comprehensive Antibiotic Resistance Database), with default parameters, respectively. The complete genome sequence has been deposited in GenBank under GenBank accession PZ791701.

### 2.8 Stability assays

To evaluate the practical applicability of vB_VhaM_HVS-1, the stability of the phage under various environmental conditions was assessed. For thermal stability, phage aliquots (1 × 10^8^ PFU/mL) were incubated at 4 °C, 25 °C, 30 °C, 37 °C, 42 °C, 50 °C, 60 °C, and 70 °C for 1 h, and the remaining phage titer was determined by the double-layer agar method. For pH stability, phage suspensions were incubated in 2216E broth adjusted to pH 3–11 using HCl or NaOH for 1 h at 28 °C, after which the phage titer was measured. For salinity tolerance, phage aliquots were incubated in 2216E broth containing 1, 2, and 3% NaCl for 1 h at 28 °C, and the phage titer was determined. For storage stability, phage suspensions were stored at 4 °C for up to 4 weeks, and the phage titer was measured weekly. All experiments were performed in triplicate (*n* = 3). Relative activity (%) was calculated as the ratio of the phage titer under each condition to the titer under optimal conditions.

### 2.9 Motility analysis

The bacterial motility analysis was measured as previously described by Wang et al. (32). The *V. harveyi* was cultured overnight and diluted 1:100 in fresh 2216E broth. Five microliters of the culture (OD₆₀₀ = 0.2 and 1.0) were spotted onto 2216E plates containing 0.4% (w/v) agar. The plates were incubated at 28 °C for 24 h, and the colony size of the motility circles was measured, respectively. All the experiments were repeated for three times.

### 2.10 TEM and SEM analyses of *V. harveyi*

The morphology of all *V. harveyi* strains were analyzed using transmission electron microscopy (TEM) mainly with the steps described below. Dilutions of *V. harveyi* stock (approximately 5 × 10^7^ CFU/mL) were deposited on carbon film and stained with 2% uranyl acetate. *V. harveyi* samples were observed using a Philips EM 300 electron microscope operated at an acceleration voltage of 120 kV at Ningbo University (Ningbo, China). Next, the recovered cultures were washed twice with PBS buffer and fixed with 2.5% precooling glutaraldehyde at room temperature for 2 h in the dark. The cells were washed twice with PBS buffer and then dehydrated with an increasing ethyl alcohol gradient (15, 30, 40, 50, 70, 100% v v^−1^) for 15 min for each step. Afterward, the cells were dried overnight, and the results were obtained through scanning electron microscopy (SEM) with an accelerating voltage of 20 kV in Ningbo University of China.

### 2.11 Effects of phages on microcolony formation and initial bacterial attachment

To quantify the effect of phages on initial bacterial attachment and microcolony formation of the *V. harveyi*, 5 μL of diluted log-phase cultures (10^7^ CFU/mL) was filtered onto 3 replicate 20 mm diameter, 0.22 μm pore size polycarbonate filters and incubated on 2216E agar in 6 well plates (33). Phage stock (10^8^ PFU/mL, 2 mL) was added, and controls with addition of 2 mL SM buffer (0.01% gelatin, 100 mM NaCl, 50 mM Tris-Cl [pH 7.5], 8 mM MgSO4). Duplicate filters were collected every 12 h for 24 h, transferred to a 20 mm diameter filtration manifold, and stained with 0.5% SYBR gold for 15 min (Invitrogen), followed by 3 rinses with PBS buffer. The filters were then quantified by epifluorescence microscopy.

### 2.12 Artificial infection and histopathological observation

The *A. japonicus* (sea cucumbers) infection experiments were conducted as described by Zhang et al. (34). Healthy young sea cucumbers (weight, 2.5 ± 0.5 g) were purchased from Dalian Pacific Aquaculture (Dalian, China) and reared in aerated natural seawater (temperature, 16 ± 1 °C; salinity, 28 ± 2) for 3 d. Then, the young sea cucumbers were randomly divided into 5 groups with 15 individuals in each group: a PBS control group and four bacterial concentration groups (4.6 × 10^4^, 4.6 × 10^5^, 4.6 × 10^6^, and 4.6 × 10^7^ CFU/mL). Each group was housed in a separate aerated tank *V. harveyi* strain NB-3 was cultured to an OD_600_nm of 1.0 in 2216E medium culture (28 °C, 12 h), centrifuged at 7000 × g for 10 min, washed with PBS and resuspended in filtered seawater. For artificial infection, weight-matched young sea cucumbers were continuously immersed in the respective bacterial suspensions. The daily mortality and incidence of skin ulceration syndrome were recorded for 7 days. Dead individuals were removed immediately, and symptoms were documented. Skin ulceration syndrome was scored as the appearance of any visible ulcerative lesion on the body wall. Humane endpoints were defined as ulceration covering >30% of the body surface or loss of response to gentle mechanical stimulation, at which point animals were euthanized by immersion in ice water (0–4 °C). All experiments were performed with random group assignment, and observations were conducted blindly by two independent observers. Complete ethics approval information is provided in the Ethics Statement section. For the ex vivo coelomocyte apoptosis assay, a separate experiment was performed with three groups (PBS control, bacteria-only, and bacteria-plus-phage) using 5 animals per group.

Body wall tissue (0.4 × 0.4 × 0.4 cm) from the sea cucumbers was fixed in 4% Paraformaldehyde in phosphate-buffered saline (PBS, pH 7.4) for 24 h, Paraffin-embedded body wall tissue and cut into 5-μm slices. The tissue sections were stained with hematoxylin for 8–10 min, followed by differentiation in 1% hydrochloric acid/alcohol for 15 s, washing in tap water for 15 min, dehydration in 95% alcohol for 30 s, and counterstaining with eosin for 15–30 s. Images were acquired using a Nikon Eclipse E100 microscope (Japan). Histopathological evaluation was performed blindly by two independent observers who were unaware of the group assignments. A semi-quantitative scoring system was applied to assess four parameters: epithelial integrity, tissue disorganization, inflammatory cell infiltration, and edema. Each parameter was graded on a scale of 0 (normal) to 3 (severe), and the total pathology score (range 0–12) was calculated as the sum of the four individual scores. Statistical comparison of scores between groups was performed using one-way ANOVA with Tukey’s *post hoc* test.

### 2.13 Coelomocyte culture

Coelomocytes were collected from healthy sea cucumbers as described in a previous work (25). Briefly, the harvested coelomocyte fluid was resuspended in Leiboviz’s L-15 medium (Invitrogen, USA) with 0.39 M NaCl at a final concentration of 1.0 × 10^6^ cells·mL^−1^. The 500 μL cell suspensions were then divided into a 24-well microplate. The cells were cultured at 16 °C for 12 h. On the other hand, *V. harveyi* was separately cultured to OD_600_nm of approximately 0.5, and centrifuged at 8000 × g for 2 min, washed and resuspended in PBS. Then, 100 μL cell suspensions of *V. harveyi* was separately added into each well of 24-well microplate, after incubation for 4 h, the non-adherent cells were washed away. Then, the adhered cells were eluted from each well of the plate with PBS containing 0.1% tween-20. The eluent was diluted in a gradient, and 50 μL of each sample was plated on 2216E plate. Number of colonies on the plate representing the adhered cells was counted, and the adhered rate was calculated based on the adhered cell number divided by the initial added cells.

### 2.14 Adherence assay

When the coelomocyte monolayer was established (1 × 10^6^ cells/mL), 100 μL of GFP tagged *V. harveyi* (1 × 10^7^ cells/mL) with an OD_600_ of approximately 0.2 was added at an MOI of 1 together with phage vB_VhaM_HVS-1 (1 × 10^7^ PFU/mL, MOI = 1) simultaneously and incubated for 3 h. After incubation, non-adherent bacteria were removed by centrifugation at 800 rpm for 3 min, followed by three washes with PBS (10 min each). The cells were then fixed with 2.5% glutaric dialdehyde (Solarbio, China) and stained with DAPI (Beyotime, China). Bacterial association with coelomocyte was visualized using a laser scanning spectral confocal microscope (TCS SP2; Leica, Solms, Germany). For quantification, GFP-positive bacteria associated with DAPI-stained coelomocytes were counted in at least five randomly selected fields per sample using ImageJ software (NIH, Bethesda, MD, USA). The adhesion rate was calculated as the number of adherent bacteria per coelomocyte divided by the total bacteria added. All experiments were performed with three independent biological replicates, each containing three technical replicates.

### 2.15 Cell apoptotic assay

*In vivo* cell apoptosis functions were assessed by flow cytometry analysis. After sea cucumbers were challenged with *V. harveyi* NB-3 for 12 h, the apoptosis rate of the collected coelomocytes was measured by FACScan (Becton Dickinson Biosciences) using an Annexin-V FITC apoptosis detection kit (Beyotime). Sea cucumbers without any treatment were used as the blank group, and sea cucumbers treated with 2216E or PBS were used as the negative control group. Briefly, the collected coelomocytes were resuspended in 195 μL Annexin V-FITC binding buffer at a final concentration of 10^6^ cells·mL^−1^ and then mixed with 5 μL Annexin V-FITC and 10 μL PI successively. After the mixture was incubated at 25°C for 10 min, the samples were analyzed by FACScan. For flow cytometry analysis, cells were first gated on FSC-A/SSC-A to exclude debris, followed by FSC-A/FSC-H gating to exclude doublets. Compensation was performed using single-stained controls (Annexin V-FITC only and PI only). A minimum of 10,000 events were acquired per sample. Instrument settings were as follows: FACScan flow cytometer (Becton Dickinson) equipped with a 488 nm argon laser, with FITC detected at 530 nm and PI at 585 nm.

### 2.16 Statistical analysis

All experiments were performed with three independent biological replicates, each containing three technical replicates. Normality of data distribution was assessed using the Shapiro–Wilk test. Statistical analyses were performed using GraphPad Prism software (version 8.0, GraphPad Software, San Diego, CA, USA). Comparisons between two groups were analyzed using Student’s t-test, while multiple group comparisons were evaluated using one-way ANOVA followed by Tukey’s *post hoc* test. All data are presented as mean ± standard deviation (SD), and differences were considered statistically significant at *p* < 0.05. Confidence intervals (95% CI) are reported where applicable. Specific *p* values are indicated in the figure legends.

## 3 Result

### 3.1 Isolation and identification of pathogen bacterial in diseased *A. japonicus*

**Figure 1:**
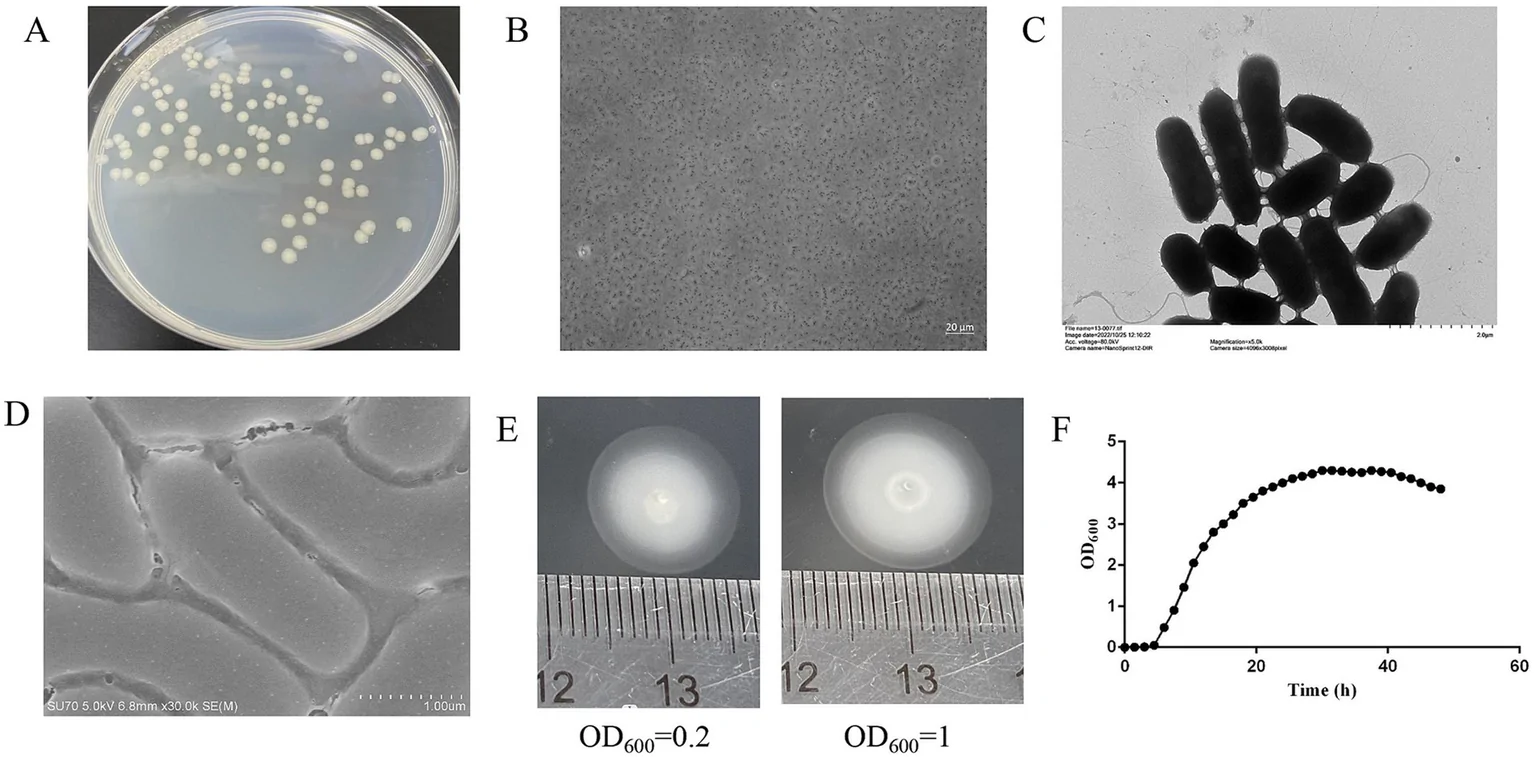
Morphology, characteristics and phage inhibition of *V. harveyi NB-*3. **(A)** Colony morphology of *V. harveyi NB-*3 on 2216E agar plate. **(B)** Visualization by phase-contrast microscopy of *V. harveyi NB-*3. **(C)** TEM analysis of *V. harveyi NB-*3. **(D)** SEM analysis of *V. harveyi NB-*3. **(E)** Swimming motility of *V. harveyi at* two different cell densities. **(F)** Growth curve of NB-3 cultured in 2216E medium at 28 °C in 2216E agar plate. Data are presented as mean ± SD (*n* = 3). Statistical significance was determined using Student’s *t*-test. *p < 0.05, **p < 0.01, ***p < 0.001, ns = not significant.

A bacterial strain which designated NB-3, was isolated from the body wall of moribund *A. japonicus* with skin ulceration syndrome in Dalian, China. The colonies of this isolated strain appeared circular, smooth, and whiteish on 2216E agar plates (Figure 1A). Phase-contrast microscopy revealed that this strain was a Gram-negative, short and curved rod-shaped (Figure 1B). Transmission electron microscopy (TEM) further confirmed the typical rod shape with a single polar flagellum (Figure 1C), while scanning electron microscopy (SEM) showed smooth and intact cell surfaces (Figure 1D). The swimming motility of *V. harveyi* NB-3 was assessed at two different cell densities (OD_600_ = 0.2 and 1.0). As shown in Figure 1E, the motility zone diameter was larger at the higher cell density. The growth curve results indicated that NB-3 displayed a typical microbial growth pattern, including early exponential phase, middle exponential phase, late exponential phase and stationary phase (Figure 1F).

### 3.2 General features of *V. harveyi* NB-3 genome

**Figure 2:**
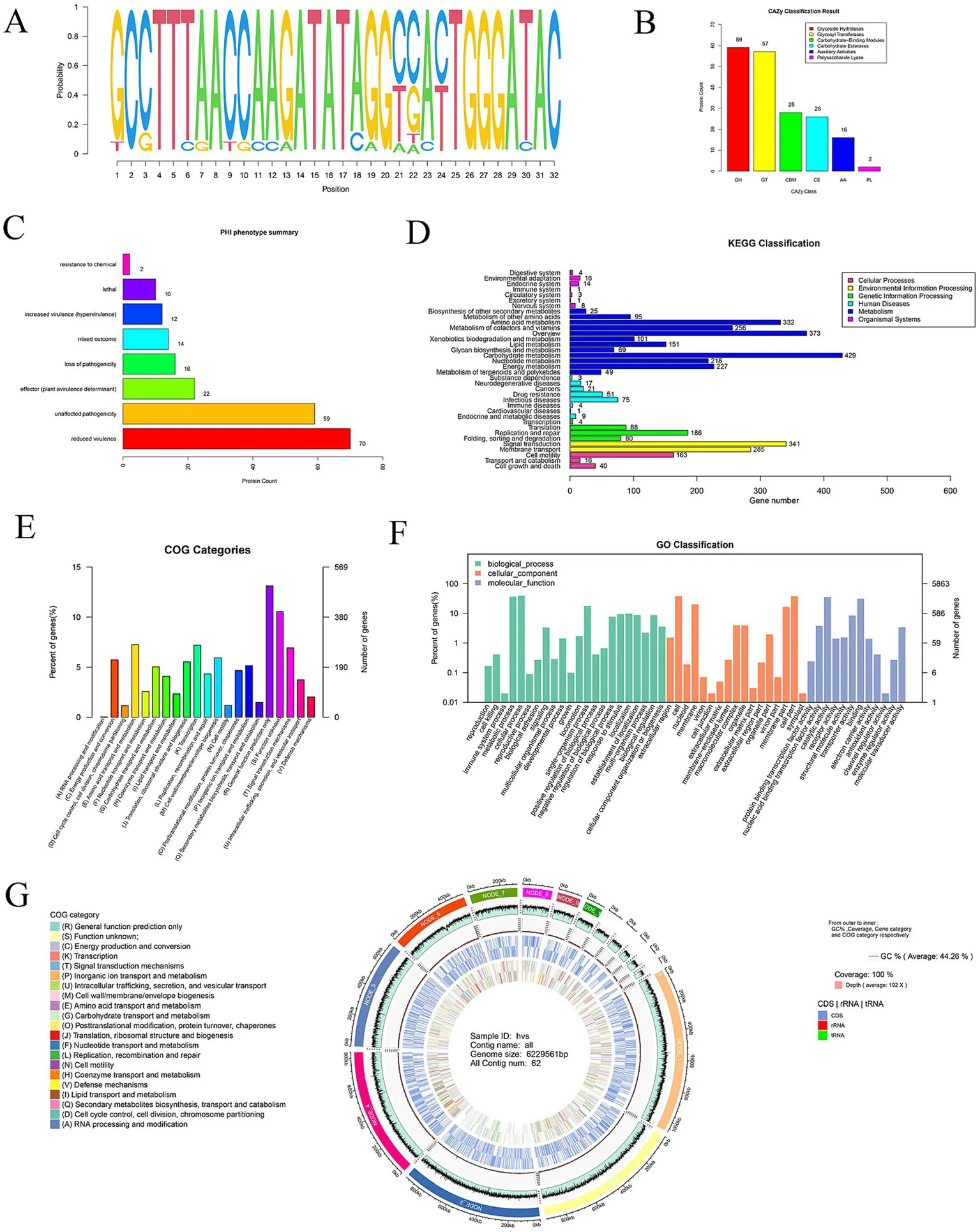
Genomic features of *V. harveyi NB-*3. **(A)** SeqLogo image of the CRISPR repeated fragments. **(B)** Functional classification statistical bar chart of CAZy (Carbohydrate-Active enZYmes Database), the horizontal axis represents the abbreviation of CAZy’s six major functional categories, the vertical axis represents the number of sequences in each category, and the caption provides explanations for the functional classifications. **(C)** Summary bar chart of pathogen host interaction (PHI) phenotypes. The vertical axis represents the nine phenotypes of pathogen host interaction, and the horizontal axis represents the number of genes annotated under each phenotype. **(D)** Histogram based on KEGG pathway classification. The horizontal axis represents the name of the metabolic pathway, and the vertical axis represents the number of genes annotated in that pathway. **(E)** Histogram based on COG classification. Each color on the horizontal axis represents a COG functional category, and the vertical axis represents the number of genes annotated under that category. **(F)** Histogram based on GO annotation. The horizontal axis represents the secondary classification of GO, and the vertical axis represents the number of genes (right) and the percentage of total annotated genes (left) within each category. Different colors represent different methodological categories. **(G)** Circular graphical map of *V. harveyi NB-*3 genome. From outer to inner rings: GC%, coverage, gene category, and COG category.

Genome sequencing was conducted on *V. harveyi* NB-3, and raw sequencing reads were assembled and annotated using multiple bioinformatics databases. The genome size, average GC content, total number of predicted genes, repeat ratio, and proportion of protein-coding sequences (CDSs) were statistically analyzed (Figure 2). The circular genome map of NB-3 is shown in Figure 2G. From the outer to the inner rings, it sequentially displays the GC distribution, sequencing coverage, gene category, and COG category information, which intuitively reflects the overall genomic structure and genetic composition of the strain (Figure 2G).

For detailed genomic analysis, the CAZy (Carbohydrate-Active enZYmes Database), PHI-base (Pathogen Host Interactions Database), GO (Gene Ontology), and KEGG (Kyoto Encyclopedia of Genes and Genomes), COG (Clusters of Orthologous Groups of proteins), and CRISPR (clustered regularly interspaced short palindromic repeat sequences) analyses were performed.

CRISPR analysis identified a clustered regularly interspaced short palindromic repeat sequence. The SeqLogo image of CRISPR repeated fragments of *V. harveyi* is shown in Figure 2A. CAZy classification results showed that glycoside hydrolases and glycosyl transferases accounted for the largest proportion of annotated sequences (Figure 2B). PHI phenotype summary identified numerous genes with sequence similarity to those associated with reduced virulence in other pathogens (Figure 2C). KEGG pathway analysis revealed that carbohydrate metabolism, amino acid metabolism, signal transduction and membrane transport pathways were among the most highly represented functional categories (Figure 2D). COG classification histogram showed that transcription and amino acid transport and metabolism were the dominant functional groups of *V. harveyi* NB-3 (Figure 2E).

GO annotation classified genes into three categories including biological process (BP), cellular component (CC) and molecular function (MF). Perform GO classification on the obtained genes, and count the GO terms of each gene in three categories of BP, CC and MF shown in Figure 2F. Cellular process and metabolic process in BP, cell and cell part in CC, catalytic activity and binding in MF were the terms of the most percent genes of *V. harveyi* NB-3 (Figure 2F).

### 3.3 Pathogenicity of *V. harveyi* NB-3 and the protective effect of phage vB_VhaM_HVS-1 on *A. japonicus*

**Figure 3:**
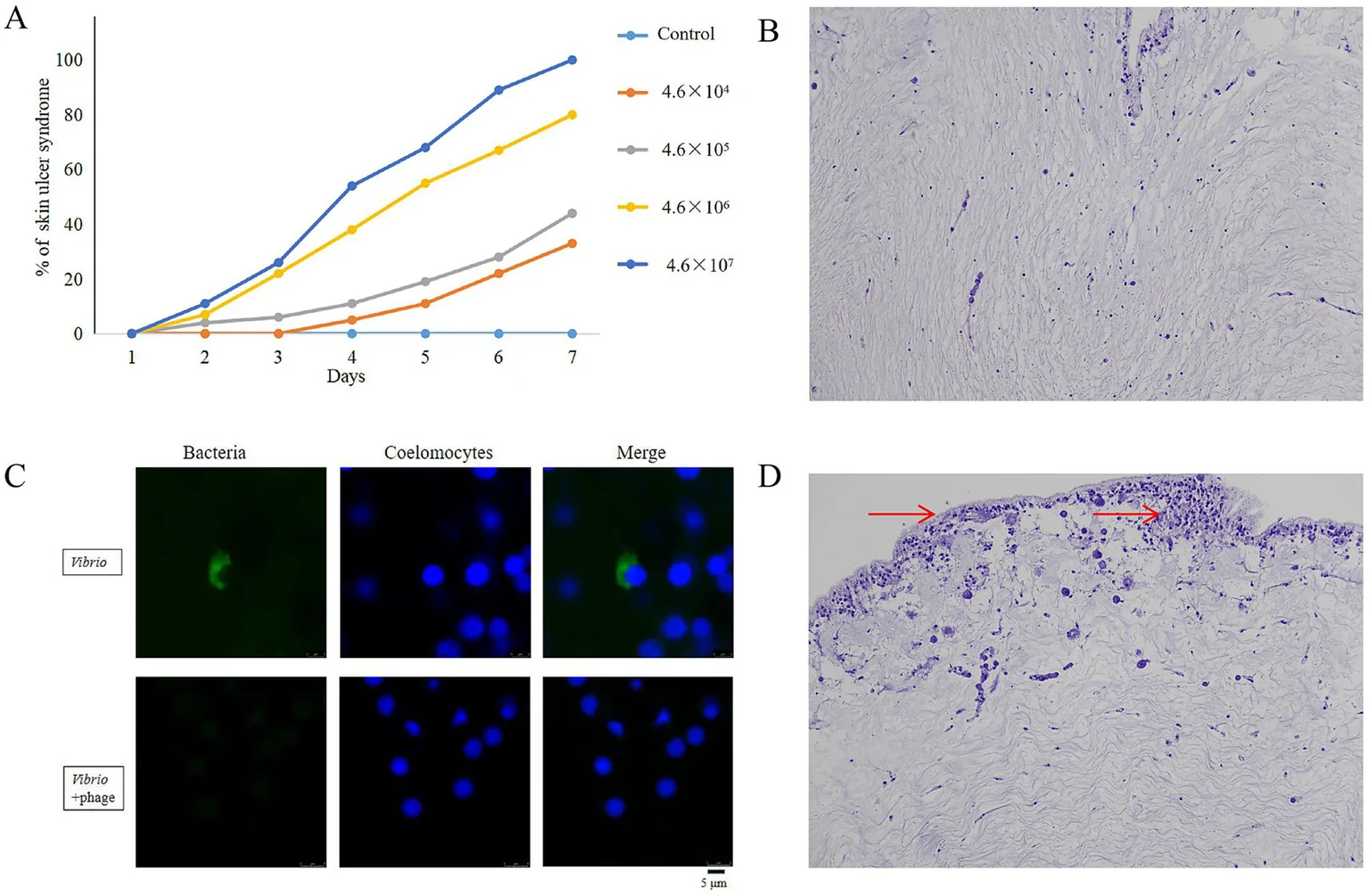
Pathogenicity of *V. harveyi NB-*3 and the protective effect of phage vB_VhaM_HVS-1 on *A. japonicus*. **(A)** Incidence of skin ulceration syndrome in sea cucumbers challenged with different concentrations of *V. harveyi*. **(B)** Histological section of sea cucumber body wall from the control group. **(C)** Confocal fluorescence microscopy images showing bacterial association to coelomocytes (upper: *V. harveyi* alone; lower: *V. harveyi* + phage; green: bacteria, blue: coelomocyte nuclei). **(D)** Histological section of sea cucumber body wall from the *V. harveyi* infected group, showing epithelial damage and inflammatory cell infiltration (red arrows). Data are presented as mean ± SD (*n* = 3). Statistical significance was determined using one-way ANOVA with Tukey’s *post hoc* test. *p < 0.05, **p < 0.01, ***p < 0.001, ns = not significant.

To evaluate the pathogenicity of *V. harveyi* NB-3, *A. japonicus* were challenged with different concentrations of NB-3 from 4.6 × 10^4^ to 4.6 × 10^7^ CFU/mL, and the incidence of skin ulceration syndrome (SUS) was recorded daily for 7 days (Figure 3A). As shown in Figure 3A, no SUS was observed in the control group (PBS) throughout the experiment. In contrast, the incidence of SUS increased in a dose and time-dependent manner. At the highest concentration (4.6 × 10^7^ CFU/mL), the incidence reached 100% by day 7, while the lowest concentration (4.6 × 10^4^ CFU/mL) resulted in only mild symptoms with low incidence, indicating the strong pathogenicity of NB-3. Bacterial reisolation from the body wall of experimentally infected animals that developed skin ulceration yielded colonies morphologically identical to NB-3. 16S rRNA sequencing confirmed the reisolates as *V. harveyi*, and no other dominant bacterial species were detected on the isolation plates.

Histopathological analysis of the body wall was performed to assess tissue damage caused by NB-3 infection. In the control group, the body wall exhibited intact tissue structure with well-organized epithelial layers and no inflammatory cell infiltration (Figure 3B). However, in the infected group, the body wall showed severe pathological changes, including epithelial cell detachment, tissue disorganization, extensive inflammatory cell infiltration, and tissue edema (Figure 3D, arrows). These lesions are consistent with the clinical symptoms of SUS, confirming that NB-3 directly induces host tissue damage. Quantitative histopathological scoring revealed that the infection group had significantly higher total pathology scores (mean ± SD: 8.5 ± 1.2) compared to the control group (1.2 ± 0.5, *p* < 0.001), while the phage-treated group showed significantly reduced scores (3.8 ± 0.9, *p* < 0.01 vs. infection group), indicating that phage treatment alleviated tissue damage.

To investigate whether phage vB_VhaM_HVS-1 could inhibit bacterial association with sea cucumber coelomocytes, a confocal fluorescence microscopy assay was performed (Figure 3C). In the group treated with *V. harveyi* alone, a large number of obvious green fluorescence signals corresponding to bacterial cells were observed around coelomocytes, indicating strong bacterial association. In contrast, the fluorescence signal was significantly reduced in the phage-treated group (*V. harveyi* + phage), and few bacteria were associated with coelomocytes. These results demonstrate that phage vB_VhaM_HVS-1 significantly reduces the number of *V. harveyi* associated with host cells.

### 3.4 Morphology, biological characteristics, and lytic activity of isolated phage

**Figure 4:**
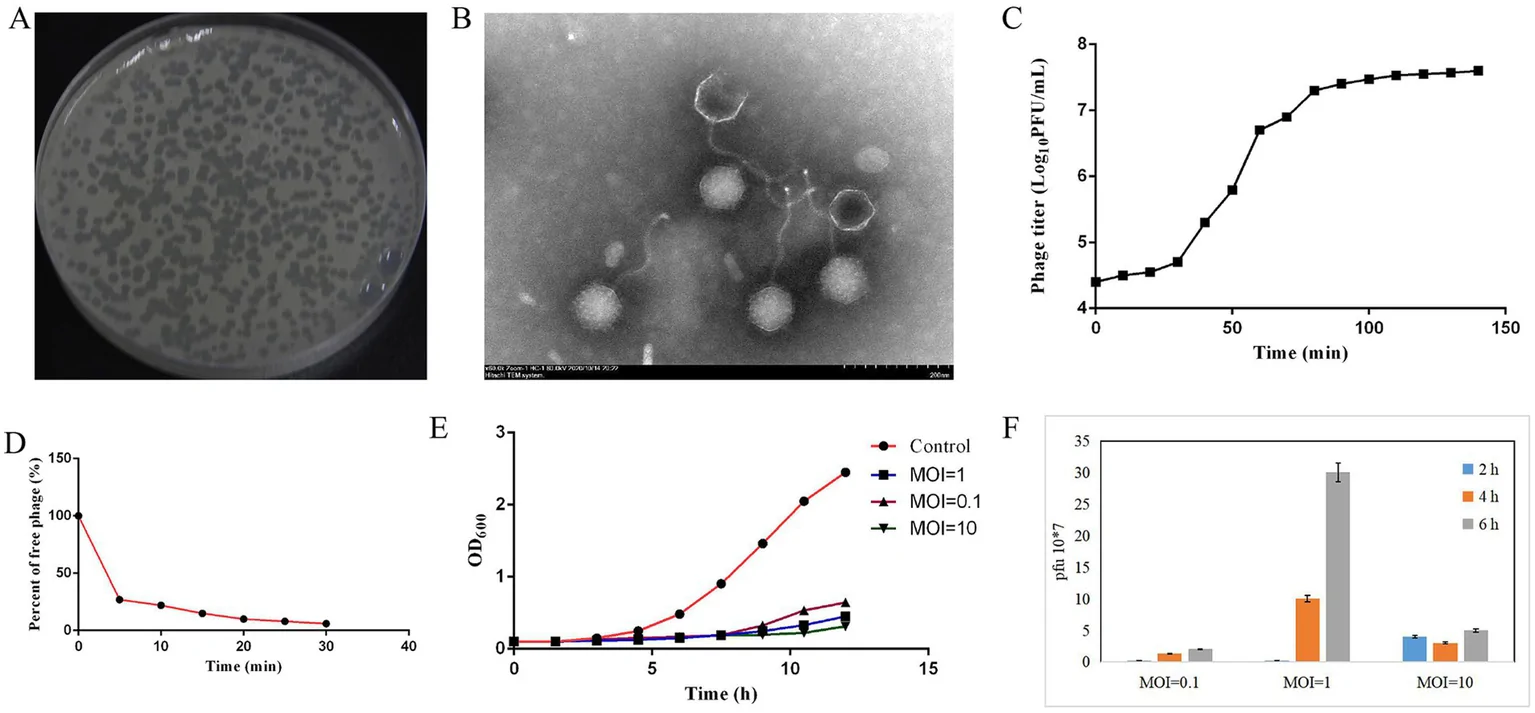
**(A)** Plaque morphology of phage vB_VhaM_HVS-1 on *V. harveyi NB-*3 lawn. **(B)** TEM of phage vB_VhaM_HVS-1. **(C)** One-step growth curve of phage vB_VhaM_HVS-1. **(D)** Adsorption rate of phage vB_VhaM_HVS-1 to *V. harveyi NB-*3. **(E)** Growth inhibition of *V. harveyi NB-*3 by phage at different MOIs. **(F)** Phage titer at different MOIs and time points post-infection. Data are presented as mean ± SD (*n* = 3). Statistical significance was determined using one-way ANOVA with Tukey’s post hoc test. *p < 0.05, **p < 0.01, ***p < 0.001, ns = not significant.

The phage vB_VhaM_HVS-1 was isolated and purified from *A. japonicus* breeding pond silt in Dalian, China. Clear and uniform plaques were formed on the bacterial lawn of *V. harveyi* NB-3 after overnight incubation at 28 °C (Figure 4A). TEM revealed that vB_VhaM_HVS-1 possesses an icosahedral head and a long contractile tail, a morphology characteristic of myoviruses (formerly classified as family *Myoviridae*). Based on its morphological features and genomic characteristics, vB_VhaM_HVS-1 is classified within the class *Caudoviricetes*, family *Straboviridae* (Figure 4B). The one-step growth curve of phage vB_VhaM_HVS-1 was determined. The latent period was approximately 50 min, followed by a rapid rise period, and the burst size was calculated to be about 168 PFU/cell (Figure 4C). The adsorption assay showed that more than 85% of the phages were adsorbed to host cells within 5 min, and nearly all phages were adsorbed by 30 min (Figure 4D).

The lytic activity of the phage against *V. harveyi* NB-3 at different multiplicities of infection (MOI) was evaluated (Figure 4E). Compared with the untreated control group, the addition of phage significantly inhibited the growth of *V. harveyi* NB-3, and the inhibitory effect was more pronounced at higher MOIs. The phage titer increased rapidly after infection and reached the highest value at 6 h post-infection at an MOI of 1 (Figure 4F).

### 3.5 Stability of phage vB_VhaM_HVS-1

**Figure 5:**
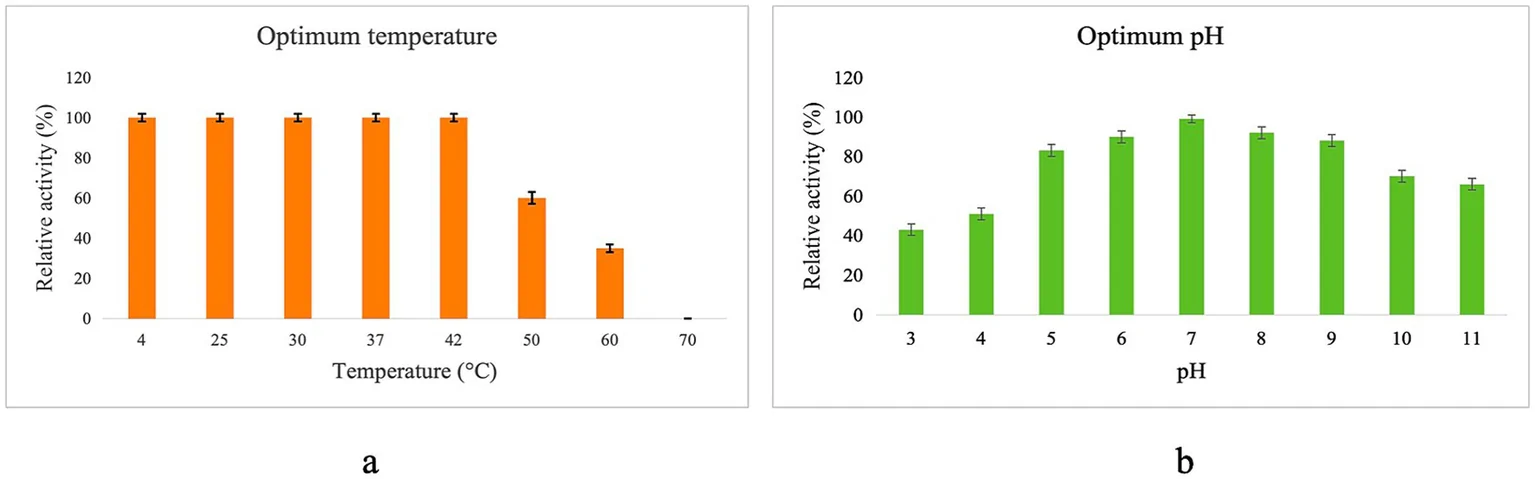
Stability of phage vB_VhaM_HVS-1 under different environmental conditions. **(a)** Thermal stability at different temperatures after 1 h incubation. **(b)** pH stability across pH 3–11. The values shown correspond to the means and SD of three independent biological repeats (*n* = 3). The values shown correspond to the means and SD of three independent biological repeats (*n* = 3). Statistical significance was determined using one-way ANOVA with Tukey’s post hoc test. *p < 0.05, **p < 0.01, ***p < 0.001, ns = not significant.

To evaluate the practical applicability of vB_VhaM_HVS-1 in aquaculture settings, the stability of the phage under various environmental conditions was assessed. Thermal stability assays showed that the phage maintained 100 ± 2% activity at 4–42 °C, with activity decreasing to 60 ± 3% at 50 °C, 35 ± 2% at 60 °C, and complete inactivation at 70 °C (Figure 5a). pH stability tests revealed that the phage was highly active across pH 5–9, with optimal activity at pH 7 (99%), and retained moderate activity at pH 10 (70%) and pH 11 (66%) (Figure 5b). The phage remained stable across a salinity range of 1–3%, with no significant loss of activity. Storage stability tests showed that the phage maintained full activity for at least 4 weeks at 4 °C. These results indicate that vB_VhaM_HVS-1 possesses favorable stability characteristics for potential application in aquaculture environments.

### 3.6 Genomic analysis of phage vB_VhaM_HVS-1

**Figure 6:**
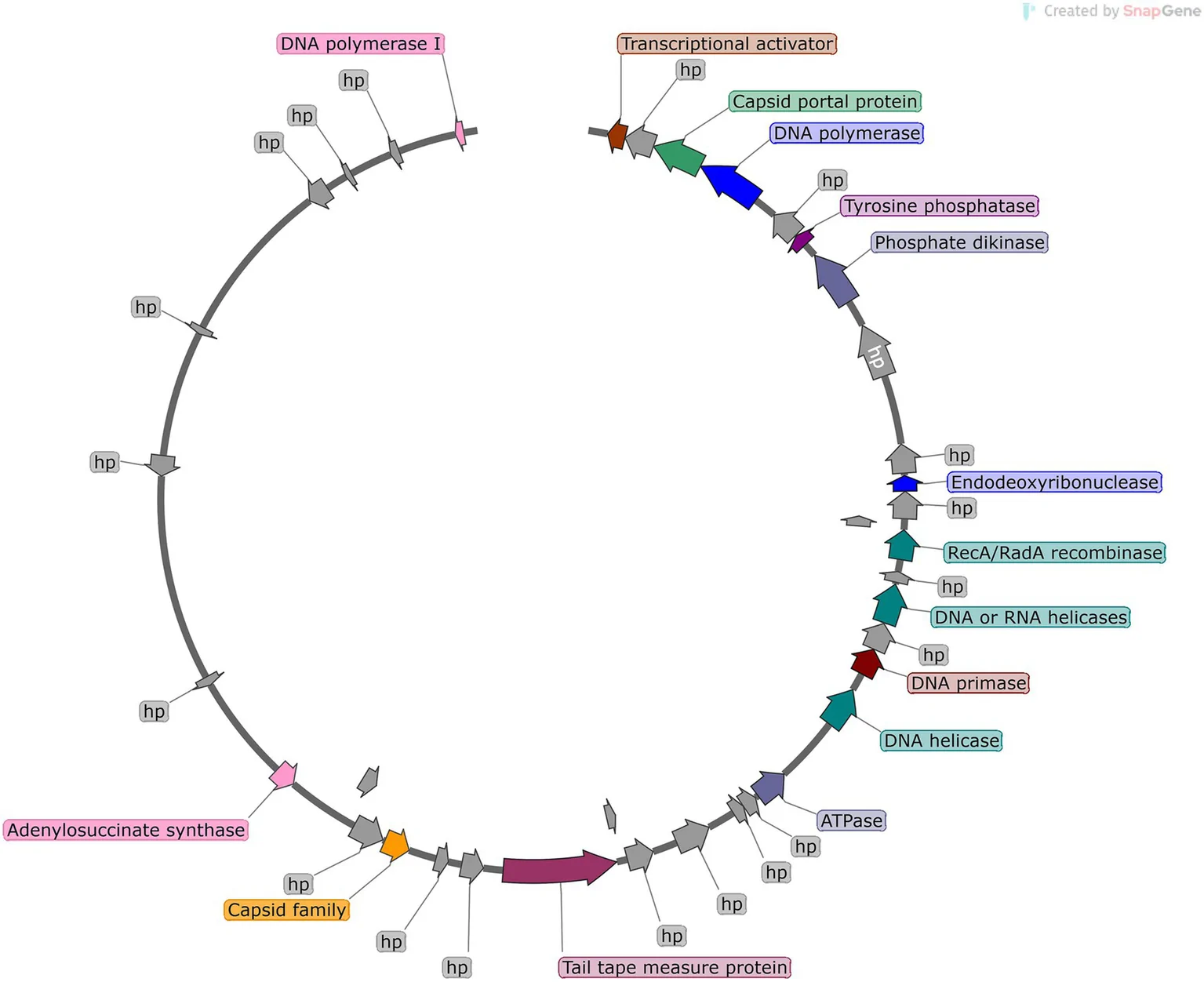
Genome organization of phage vB_VhaM_HVS-1. The direction of the arrow indicates the direction of transcription, hp. represents of hypothetical protein.

The complete genome of phage vB_VhaM_HVS-1 was sequenced and functionally annotated (Figure 6). The genome is a double-stranded DNA molecule of 81,118 bp assembled as a single contig (N50 = 81,118 bp), with a GC content of 45.62%. A total of 110 protein-coding genes were predicted, with an average length of 686.56 bp and a coding ratio of 93.10%. No repeat regions were detected in the genome. Functional annotation against COG, KEGG, and GO databases revealed that the majority of annotated genes are involved in DNA replication, recombination, and repair (50%), transcription (10%), carbohydrate transport and metabolism (10%), and signal transduction mechanisms (10%). The circular genomic map displays predicted ORFs encoding structural proteins (capsid family, portal protein, tail tape measure protein), DNA replication/metabolism-related proteins (DNA polymerase, helicase, primase, recombinase), and a transcriptional activator, alongside numerous hypothetical proteins (hp). Comprehensive annotation against VFDB (Virulence Factors Database), CARD (Comprehensive Antibiotic Resistance Database), and toxin-related databases confirmed the absence of known virulence genes, toxin genes, and antimicrobial resistance genes in the genome. No lysogeny-related genes were detected, suggesting its lytic nature. The complete genome sequence has been submitted to NCBI GenBank (GenBank accession PZ791701).

### 3.7 Phage vB_VhaM_HVS-1 inhibits bacterial adhesion and protects coelomocytes from *V. harveyi* infection

**Figure 7:**
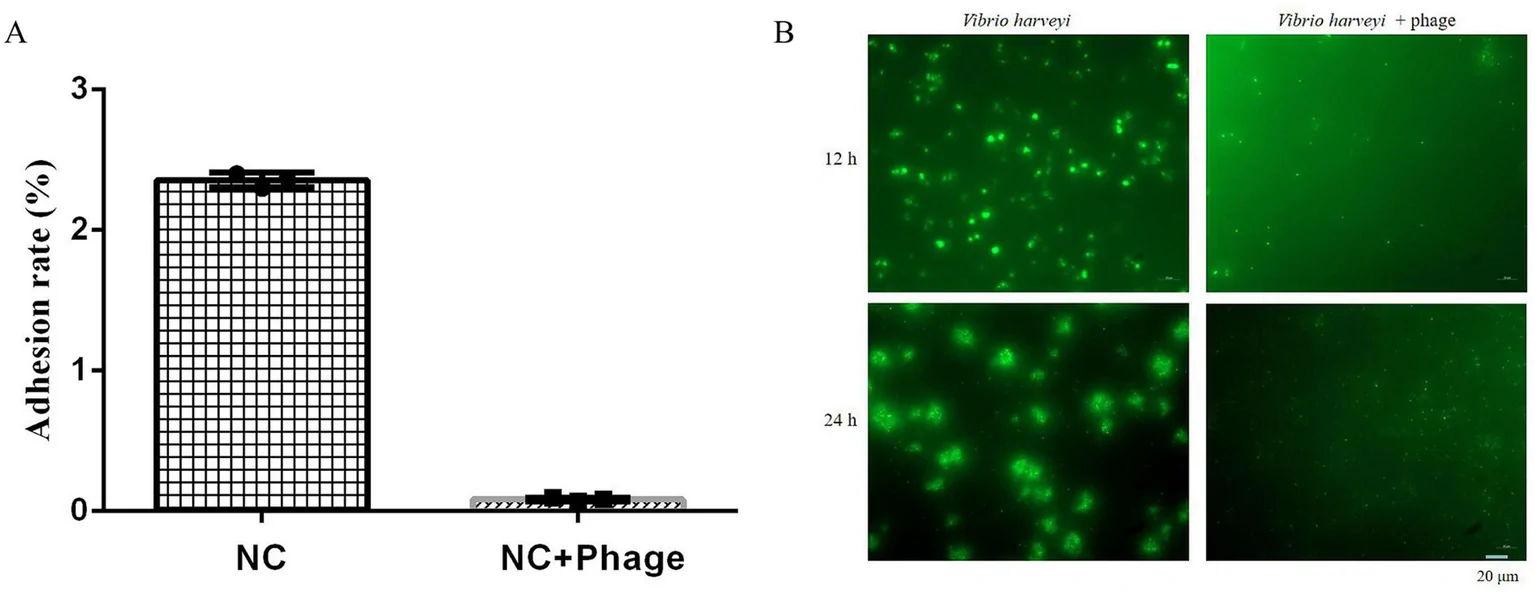
**(A)** Quantitative comparison of bacterial association rates between the *V. harveyi* infection group (NC, without phage) and phage-treated group (NC + Phage, *V. harveyi* + phage). **(B)** Fluorescence microscopy images showing bacterial adhesion to host cells at 12 h and 24 h post-infection (left: *V. harveyi*; right: *V. harveyi* + phage). Data are presented as mean ± SD (*n* = 3). Statistical significance was determined using Student’s *t*-test. *p < 0.05, **p < 0.01, ***p < 0.001, ns = not significant.

To assess the effect of phage vB_VhaM_HVS-1 on bacterial association with host coelomocytes, an adhesion assay was performed (Figure 7A). The percentage of coelomocytes associated with bacteria in the untreated control group was approximately 2.3%, while the percentage was reduced to nearly zero in the phage-treated group, indicating a highly significant reduction in cell-assionciated bacterial following phage treatment. Fluorescence microscopy further confirmed this effect (Figure 7B). At both 12 h and 24 h post-infection, abundant green fluorescence signals corresponding to adherent bacteria were detected in the *V. harveyi* group, whereas only weak or almost no fluorescence signals were observed in the phage co-treated group.

**Figure 8:**
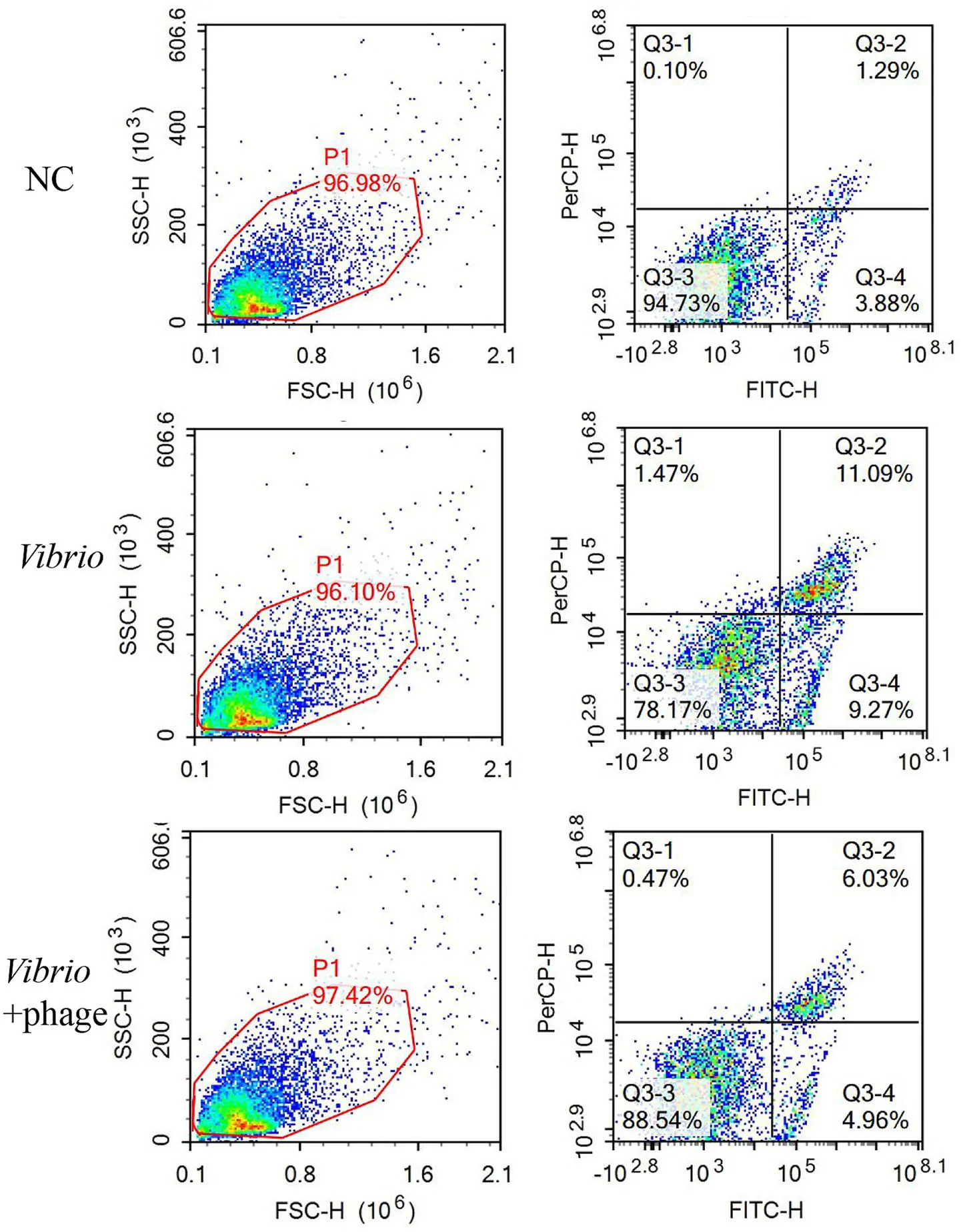
Flow cytometry analysis of the changes in coelomocyte apoptosis at 12 h after *V. harveyi* infection (NC) with or without phage vB_VhaM_HVS-1. NC represents the *V. harveyi* infection group (without phage). Data are presented as mean ± SD (*n* = 3). Statistical significance was determined using Student’s *t*-test. *p < 0.05, **p < 0.01, ***p < 0.001, *ns* = not significant.

In addition, the cytoprotective effect of the phage was further verified by flow cytometry (Figure 8). In the negative control (NC) group, the proportion of viable host cells (Q3-3) was 94.73%, with very few apoptotic or necrotic cells. Following infection with *V. harveyi*, the proportion of viable cells decreased to 78.17%, and the proportion of apoptotic and necrotic cells increased significantly. However, in the *V. harveyi* + phage co-treatment group, the viable cells recovered to 88.54%, and the proportion of damaged cells decreased substantially. These results demonstrate that phage vB_VhaM_HVS-1 effectively protects coelomocytes from *V. harveyi*-induced damage.

## 4 Discussion

In this study, a pathogenic strain *V. harveyi* NB-3 was isolated from diseased *A. japonicus* with skin ulceration syndrome. The morphological observation, physiological growth characteristics and genomic sequencing results confirmed that the isolated strain belonged to *V. harveyi*, which was consistent with previous reports that *V. harveyi* is one of the dominant pathogenic microorganisms causing mass mortality and economic losses in sea cucumber aquaculture (5, 24). NB-3 exhibits density-dependent swimming motility and carries genes associated with virulence, carbohydrate metabolism, and CRISPR defense systems (35–37). Artificial infection assays confirmed its dose- and time-dependent pathogenicity, with 100% incidence of skin ulceration syndrome at 4.6 × 10^7^ CFU/mL by day 7, and histopathological examination revealed epithelial detachment and inflammatory infiltration (38). Bacterial re-isolation from the body wall of experimentally infected animals that developed skin ulceration yielded colonies morphologically identical to NB-3, and 16S rRNA sequencing confirmed the re-isolates as *V. harveyi*, with no other dominant bacterial species detected on the isolation plates. These findings support the conclusion that NB-3 is a causative pathogen of skin ulceration syndrome in cultured *A. japonicus*, fulfilling the criteria of Koch’s postulates. However, we acknowledge that other potential pathogens were not comprehensively screened in the original diseased animals.

To combat this pathogen, a lytic phage vB_VhaM_HVS-1 was isolated from *A. japonicus* breeding pond silt. The phage belongs to the family *Straboviridae* and exhibits strong lytic activity against NB-3, with a latent period of 40 min and a burst size of approximately 100 PFU/cell. Importantly, phage treatment significantly reduced the adhesion rate of *V. harveyi* to coelomocytes from 2.3% to nearly undetectable levels and increasing the proportion of viable cells from 78.17 to 88.54%. This protective effect is likely attributed to the reduction of bacterial load, limiting host exposure to virulence factors such as lipopolysaccharide (LPS) (29, 30). LPS is a major component of the outer membrane of Gram-negative bacteria and has been shown to induce coelomocyte apoptosis in *A. japonicus* via the integrin/FAK/caspase-3 signaling pathway (29). By reducing the number of viable bacteria available for adhesion, phage treatment decreases the local concentration of LPS and other virulence factors at the host–pathogen interface, thereby reducing the apoptotic stimulus to coelomocytes. In addition, the rapid lysis of *V. harveyi* by vB_VhaM_HVS-1 may prevent the establishment of robust bacterial biofilms, which are known to enhance bacterial persistence and host colonization (39). While we cannot exclude the possibility that phage-derived components contribute directly to host cell protection, the correlation between reduced bacterial load and increased coelomocyte viability observed in this study supports the interpretation that bacterial load reduction is the primary protective mechanism.

Phage infection is initiated by the specific recognition and attachment to receptors on the bacterial surface, a process mediated by viral receptor-binding proteins (RBPs). In *Vibrio* species, the primary receptors identified for phage adsorption include LPS, capsular polysaccharide (CPS), and outer membrane proteins such as OmpK (40). The type of receptor utilized often correlates with phage morphology; for instance, myoviruses like vB_VhaM_HVS-1 frequently use LPS as a primary receptor (41). The adsorption process can involve reversible binding to a primary receptor, followed by irreversible attachment to a secondary receptor (42). Recent studies have identified specific RBPs in related phages. For example, the tail protein gp02 of the *Autographiviridae* phage HH109 was confirmed as the RBP for CPS in *V. alginolyticus* (43). Additionally, quorum sensing has been shown to positively regulate the expression of CPS-dependent phage receptors, thereby increasing phage adsorption and infection rates (44). While these detailed mechanisms remain to be elucidated for vB_VhaM_HVS-1, the strong adsorption observed in our study, combined with its myovirus morphology, suggests that its initial host recognition likely involves interactions with surface polysaccharides such as LPS or CPS. Future work will focus on identifying the specific receptor(s) and RBPs for vB_VhaM_HVS-1 through approaches like transposon mutagenesis, phage-resistant mutant selection, and RBP prediction algorithms.

The emergence of phage-resistant bacteria represents a significant challenge for the clinical application of phage therapy. In *Vibrio* species, the frequency of spontaneous resistance has been reported to range from 1.8 × 10^−6^ to 3.1 × 10^−6^ per generation (45), although this frequency can vary considerably depending on the phage-host pair and environmental conditions. Bacteria can develop resistance through multiple mechanisms, with the most common being mutations in bacterial surface receptors that phages recognize for adsorption (16). In *Vibrio parahaemolyticus*, mutation of the *flaG* gene was found to prevent phage adsorption to the host cell surface, while the resulting resistant mutants exhibited reduced growth competitiveness (46). Similarly, in *Vibrio alginolyticus*, phage-resistant mutants often harbor mutations in capsular polysaccharide biosynthesis genes that reduce phage adsorption (43). In *Vibrio alginolyticus*, acquisition of phage resistance has been shown to perturb the quorum-sensing cascade, leading to reduced expression of regulatory proteins such as LuxM, LuxN, and LuxP, and resulting in altered biofilm formation, reduced planktonic growth, and attenuated virulence (47). In addition to receptor mutations, bacteria can employ CRISPR-Cas adaptive immunity systems to resist phage infection. Given these challenges, phage cocktails combining multiple phages with distinct host ranges and receptor specificities have been proposed as an effective strategy to mitigate resistance development (17). Recent studies have demonstrated that rationally designed phage cocktails can suppress bacterial regrowth and delay the emergence of resistance more effectively than single-phage treatments (48). For future applications of vB_VhaM_HVS-1, we plan to evaluate resistance frequency, characterize the genetic basis of resistance through whole-genome sequencing of spontaneous resistant mutants, and develop phage cocktails combining vB_VhaM_HVS-1 with other lytic phages targeting distinct receptors to enhance long-term therapeutic efficacy.

To our knowledge, this is the first study to evaluate phage therapy against *V. harveyi* at the cellular level in *A. japonicus*. While several lytic phages targeting *V. harveyi* have been reported, the majority belong to the family *Siphovirida*, whereas vB_VhaM_HVS-1 belongs to the family *Straboviridae*, with a genome of 81,118 bp, distinct from the jumbo phage vB_VhaM_pir03 (286 kb, 334 ORFs) (13, 49, 50). Its genome lacks lysogeny-, virulence-, and antibiotic resistance-related genes, supporting its safety for biocontrol applications. Unlike broad-spectrum phages such as vB_VhaM_pir03, vB_VhaM_HVS-1 exhibits high specificity for *V. harveyi* NB-3, which is advantageous for precision biocontrol by minimizing off-target effects on beneficial microbiota. Previous studies in sea cucumber aquaculture have demonstrated that phage therapy can be effective against *Vibrio* species, with a phage targeting *V. cyclitrophicus* increasing juvenile survival from 18 to 81% (51), and a three-phage cocktail effectively controlling *Vibrio* infections (15, 39, 52). Our findings complement these studies by providing cellular-level mechanistic evidence for phage-mediated protection.

We acknowledge several limitations in this proof-of-concept study. Host range was tested only against the single pathogenic strain NB-3, and the phage receptor, adsorption mechanism, and receptor-binding proteins remain unidentified. Environmental stability, optimal MOI, and phage resistance frequency were not systematically evaluated. Additionally, a comprehensive *in vivo* therapeutic evaluation was not performed. The observed reduction in cell-associated bacteria following phage treatment may result from phage-mediated bacterial lysis rather than a specific anti-adhesion mechanism. Without normalization to viable bacterial concentrations or inclusion of controls such as heat-inactivated phage, we cannot distinguish between these possibilities. Moreover, the classification of vB_VhaM_HVS-1 as lytic is based primarily on genomic evidence and the presence of strong lytic activity observed in one-step growth experiments. While this approach is widely used in phage characterization studies, definitive confirmation of a strictly lytic lifestyle would require biological evidence such as failure to recover stable lysogens. Future work will address these parameters to assess the feasibility of vB_VhaM_HVS-1 for field application in *A. japonicus* aquaculture.

Despite these limitations, the biological characteristics of vB_VhaM_HVS-1 support its potential for aquaculture application. Its strictly lytic nature, absence of virulence and antibiotic resistance genes, and high host specificity make it a promising candidate for precision biocontrol in *A. japonicus* farming (13, 49, 50). However, several challenges must be addressed before field implementation. Environmental factors can significantly affect phage persistence and activity in open aquaculture systems. The narrow host range, while advantageous for precision targeting, may limit efficacy against polymicrobial infections. Practical considerations such as scalable production, cost-effective formulation, and appropriate delivery methods also require further development. Moreover, regulatory approval for phage-based products in aquaculture demands comprehensive safety and efficacy data. Addressing these challenges will be essential for translating the promising *in vitro* and ex vivo findings of this study into practical applications for the *A. japonicus* aquaculture industry.

## 5 Conclusion

This study demonstrates that phage vB_VhaM_HVS-1 significantly reduces *V. harveyi* association with coelomocytes and protects against coelomocyte apoptosis in *A. japonicus* in ex vivo assays. The phage exhibits strong lytic activity and lacks recognizable lysogeny-related genes, supporting its potential as an alternative biocontrol agent for controlling *V. harveyi* infection in sea cucumber aquaculture. Further research on *in vivo* efficacy is needed to validate its therapeutic potential for practical application.

## Data Availability

The genome sequence of *V. harveyi* NB‑3 has been deposited in the NCBI GenBank database and is publicly available under the accession number PZ791701.
